# Network Centrality Analysis Characterizes Brain Activity during Response Inhibition in Right Ventral Inferior Frontal Cortex

**DOI:** 10.14789/jmj.JMJ21-0055-OT

**Published:** 2022-04-15

**Authors:** UTA FUJIMOTO, AKITOSHI OGAWA, TAKAHIRO OSADA, MASAKI TANAKA, AKIMITSU SUDA, NOBUTAKA HATTORI, KOJI KAMAGATA, SHIGEKI AOKI, SEIKI KONISHI

**Affiliations:** 1 Department of Neurophysiology, Juntendo University School of Medicine, Tokyo, Japan; 1 Department of Neurophysiology, Juntendo University School of Medicine, Tokyo, Japan; 2 Department of Neurology, Juntendo University School of Medicine, Tokyo, Japan; 2 Department of Neurology, Juntendo University School of Medicine, Tokyo, Japan; 3 Department of Radiology, Juntendo University, School of Medicine, Tokyo, Japan; 3 Department of Radiology, Juntendo University, School of Medicine, Tokyo, Japan

**Keywords:** inferior frontal gyrus, inferior frontal junction, boundary mapping

The right inferior frontal cortex (IFC) plays a critical role in response inhibition^1, 2)^. It has also been demonstrated that the IFC is heterogeneous and that the ventral part of the IFC (vIFC) is more critical to inhibition of prepotent response tendency. Recent areal parcellation analyses based on resting-state functional connectivity have revealed that the right vIFC consists of multiple functional areas. Resting-state functional connectivity analyses have enabled parcellation of the cerebral cortex into functional areas based on their connectivity patterns^3-5)^. Parcellation analyses have revealed multiple areas (parcels) in the vIFC^3-5)^, suggesting functional heterogeneity within the vIFC. In the present study, we characterized the parcellated areas (parcels) in the right vIFC using graph-theoretic analysis, which characterizes local connectivity properties of a brain network by referring to its global structure of functional connectivity. This abstract is based on a study first reported in Neuroscience^6)^.

Twenty right-handed subjects (10 men and 10 women, aged 26.6 ± 9.2 years (mean ± SD)) participated in the experiments. The experimental procedures were approved by the Institutional Review Board. Written informed consent was obtained from all subjects. Functional magnetic resonance imaging (fMRI) scans were acquired during resting state and during the performance of a stop-signal task^1, 7)^. We used multi-band gradient-echo echo-planar sequences for functional images (TR = 1.0 sec, TE = 30 msec, flip angle = 62 deg, FOV = 192 × 192 mm^2^, matrix size = 96 × 96, 78 contiguous slices, voxel size = 2.0 × 2.0 × 2.0 mm^3^, multi-band factor = 6).

For the resting-state dataset, preprocessing was conducted mainly following the pipelines of Human Connectome Project. The parcellation analyses based on boundary mapping were applied to the cerebral cortical surface. For subsequent analyses, to avoid non-uniform signal to noise ratio caused by the different number of vertices in the parcels, we defined regions of interest (ROIs) of 40 vertices closest to the centroid of each parcel. When the parcel contained less than 40 vertices, the ROI included all the vertices in the parcel. Temporal correlations of preprocessed time-series between the ROIs were calculated as the strength of functional connectivity. A binary undirected network for each subject was defined using a proportional threshold (top 9%), and the betweenness centrality of each parcel was calculated^8-11)^. For the task dataset, we contrasted Stop success and Go success trials to reveal the brain activation for response inhibition.

In total, 377 parcels were identified in the cortical surfaces. A ROI was defined for each parcel (Figure 1A). Figure 1B shows the centrality index across the ROIs for one representative subject. The subjects performed the stop-signal task in the scanner that comprised of Go trials and Stop trials (Figure 1C). Brain activation for response inhibition was observed in several regions including the right IFC (Figure 1D). Two parcels (ROI 1 and ROI 2) in the right vIFC were significantly activated (ROI 1, t(19) = 5.42, p < 0.001; ROI 2, t(19) = 5.91, p < 0.001). There was no significant difference in the task-related activity in the ROIs [t(19) = -0.71, P = 0.49]. ROI 1 was located more ventrally, whereas ROI 2 was located more dorsally in the vIFC (Figure 2A). The correlations between the centrality and the brain activity were calculated in the two ROIs. ROI 1 showed significant correlation (r = 0.62, p = 0.003) (Figure 2B), whereas ROI 2 did not (r = -0.01, p = 0.97) (Figure 2C). The difference in the correlation was also significant (z = 2.15, p = 0.032). For ROI 1, which was significantly correlated, the correlation between activation and SSRT was significant (r = -0.50, p = 0.026) (Figure 2D). However, the correlation was not significant between centrality and SSRT (r = -0.01, p = 0.95).

**Figure 1:**
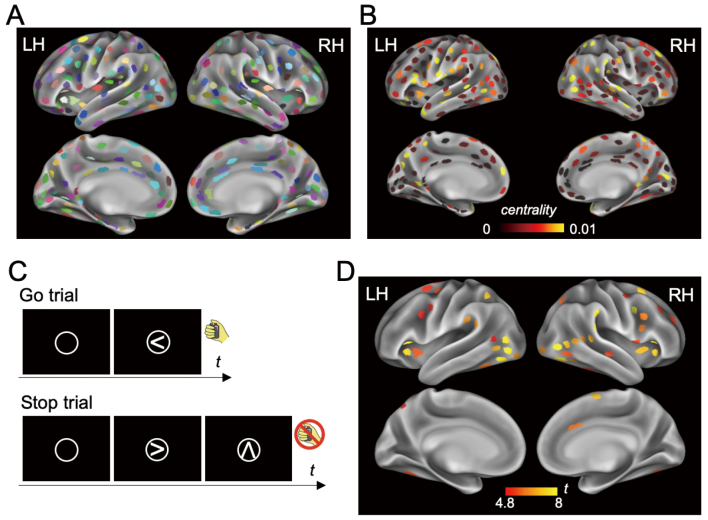
Centrality map and activation in stop signal task. **A.** ROIs defined using areal parcellation. In total, 377 ROIs were defined (192 in left hemisphere and 185 in right hemisphere). The colors are randomly selected. **B.** Centrality map on ROIs of one representative subject. **C.** Stop signal task. A circle was presented at the center of the screen as a warning. In Go trials, a left- or right-pointing arrow was presented inside the circle. The participants were instructed to press a button indicating the corresponding side. In Stop trials, an arrow was first presented inside the circle, similar to Go trials. Then, the arrow was changed to an up-pointing arrow. The participants were required to withhold the response. **D.** Activated ROIs in the vIFC (Stop success minus Go success). LH, Left hemisphere; RH, Right hemisphere. Modified from Fujimoto et al.^6)^.

**Figure 2:**
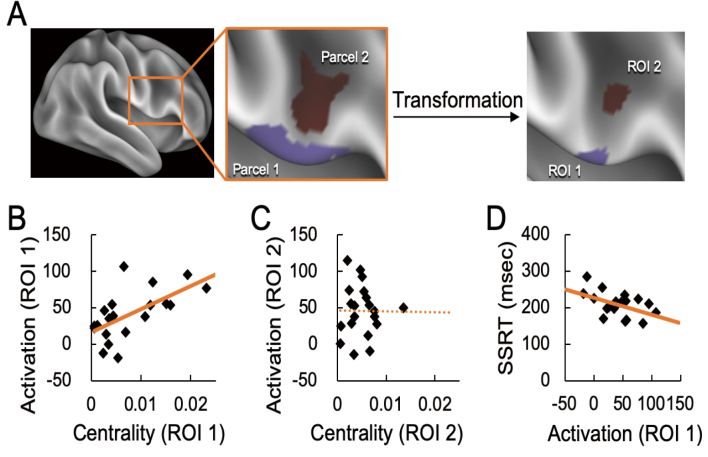
Relations among behavior, task activation, and network centrality. **A.** Target parcel ROIs in the IFC. **B.** Correlation between the centrality indices and the brain activity (Stop success minus Go success) in ROI 1. They are significantly correlated (r = 0.62, p = 0.003). **C.** Correlation between centrality indices and brain activity in ROI 2. They are not significantly correlated (r = -0.01, p = 0.97). **D.** Correlation between brain activity and SSRT in ROI 1. They are significantly correlated (r = -0.50, p = 0.026). Modified from Fujimoto et al.^6)^.

In the ventral parcel in the vIFC, the correlation between centrality and brain activity during response inhibition was significant. Whereas the correlation between brain activity and SSRT was also significant, the correlation between the centrality and SSRT was not significant in the parcel. These results suggest that the ventral part of right vIFC is involved in stopping behavior and plays a critical role in the brain network for response inhibition.

## Funding

This work was supported by JSPS KAKENHI Grant Number 19K07807 to A.O. and 18K07348 to T.O. and a grant from Takeda Science Foundation to S.K.

## Author Contributions

UF, AO, and SK designed research. AO, TO, MT, AS, NH, KK, and SA performed research. UF, AO, SK, and TO summarized data and wrote the paper.

## Conflicts of interest statement

The authors declare that there are no conflicts of interest.
